# Fracture Performance and Crack Propagation Mechanism of Basalt Fiber-Reinforced Asphalt Mixtures: Effects of Gradation, Mortar and Test Conditions

**DOI:** 10.3390/ma19122443

**Published:** 2026-06-07

**Authors:** Ziyun Fei, Keke Lou, Wentong Xu, Silin Jia, Cong Zhang, Zhengguang Wu

**Affiliations:** 1 School of Civil Engineering and Transportation, Yangzhou University, Yangzhou 225127, China; 13961740804@163.com (Z.F.); x49931933@163.com (W.X.); 18651055606@163.com (C.Z.);; 2 Research Center for Basalt Fiber Composite Construction Materials, Yangzhou 225127, China

**Keywords:** asphalt mixture, basalt fiber, semi-circular bending test, digital image correlation, crack propagation

## Abstract

To explore the fracture performance and crack propagation mechanism of basalt fiber (BF)-reinforced asphalt mixtures and overcome the limitations of single-factor performance evaluations, this study systematically investigates the effects of aggregate gradation, material scale and test conditions on fracture behavior. The semi-circular bending (SCB) test was integrated with digital image correlation (DIC) technology to synchronously obtain macroscopic fracture parameters and full-field displacement/strain fields. The findings showed that fine aggregate particle size could better utilize the bridging effect of BFs, increasing fracture energy by 25.8% versus 15.9% for the coarse aggregate particle size. A consistent enhancement in fracture performance is observed between the asphalt mixture and the asphalt mortar after BF incorporation. Under the same test conditions, the addition of fibers increased the fracture energy by 25.8% for the mixture and by 28.4% for the mortar, while fracture toughness increased by 6.9% and 8.3%, respectively. The lower loading rate reduces the reinforcement effect due to viscoelastic stress relaxation, while low temperatures enhance the relative crack resistance efficiency of BFs. The incorporation of fibers increases the crack tortuosity coefficient by a range of 4–14%, leading to greater energy dissipation. However, low temperatures absolutely dominate the crack morphology. This study provides an experimental reference for the differentiated design of BF-reinforced asphalt mixtures under different gradation types and climatic conditions.

## 1. Introduction

Asphalt mixtures are primary materials in road engineering. During service, they are constantly subjected to traffic loads, temperature changes, and water infiltration. These factors induce crack formation and expansion, weakening the durability and structural integrity of the pavement [1,2,3]. Therefore, studying the crack resistance of asphalt mixtures and their influencing factors is of great significance for improving the long-term performance.

Fibers can improve asphalt mixture crack resistance. Previous investigations concerning fiber category, dimensions and content indicate their substantial impact on the mechanical characteristics, pavement performance and fracture behavior of mortars and mixtures [4,5,6,7,8]. Basalt fibers (BFs) are widely used due to their high mechanical strength, thermal stability and interfacial compatibility. Qin et al. [4] found that BFs can form a relatively stable reinforcing structure within the material, and enhance the crack resistance of the mortar through adsorption and reinforcement effects. Wu and Hu et al. [5,7] further demonstrated that BFs can improve the crack resistance and road performance of asphalt mixtures. Additionally, Pei et al. [8] pointed out that the change in fiber diameter would significantly affect the crack propagation process and crack inhibition effect of basalt fiber–asphalt mixtures. Recent studies have explored fiber characteristic parameters [9], fiber morphology [10], spatial stress networks [11], surface modification [12], multi-factor design [13] and optimized fiber blending [14]. Collectively, these studies confirm the potential of basalt fibers in enhancing mixture crack resistance.

Regarding crack resistance evaluation, the semi-circular bending (SCB) test is a laboratory testing method based on the principles of fracture mechanics that simulates the cracking behavior of the asphalt mixtures and has been widely applied in the research on the fracture properties of materials [2,3,15]. Wang et al. [16] analyzed the effects of temperature, asphalt type and loading rate on the fracture parameters. Pirmohammad et al. [17] used fracture toughness as an evaluation index and studied the influence of BF length and dosage on crack performance. Saha et al. [18] systematically reviewed the application of the SCB test and pointed out that this method can characterize the fracture energy, flexibility index and post-peak crack propagation behavior. In addition, Yang et al. [19] extended the SCB test to asphalt mortar models and proved that this method is also applicable to evaluating the low-temperature crack resistance of mortar materials. Meng et al. [20] conducted a systematic study on the fatigue properties of large-stone asphalt mixtures based on the SCB test. Wu et al. [21] comprehensively evaluated SMA-13 asphalt mixtures reinforced by different fiber types, demonstrating that fiber type significantly influences the anti-cracking performance.

In addition to the material itself, testing conditions, such as loading rate and test temperature, also significantly affect fracture behavior [16,22]. Moreover, gradation structure and coarse aggregate skeleton characteristics also influence crack path, fracture process zone and material energy dissipation capacity. Vaseghi et al. [23] systematically analyzed the effects of SCB specimen size, loading mode and loading rate on fracture resistance indicators at different temperatures. He et al. [24] employed the Weibull distribution to predict the effect of loading rate on the fracture toughness of asphalt concrete using the SCB test. Pirmohammad [25] and Song et al. [26] investigated the size effect on fracture parameters at different temperatures. Therefore, it is difficult to fully explain the mechanism by which basalt fiber affects the crack resistance of asphalt mixtures solely from a single fracture index.

Due to asphalt’s heterogeneity and anisotropy, traditional gauges struggle to capture local crack-tip strains, necessitating non-contact optical methods for refined fracture characterization. The provision of full-field surface strain and displacement data via digital image correlation (DIC) under loading conditions enables the precise identification of crack onset, stable growth and terminal failure [27,28]. Seo et al. [28] first applied DIC to the mechanical testing of asphalt–aggregate systems and demonstrated its applicability in local displacement measurement. Buttlar et al. [27] systematically discussed the application of DIC in the study of strain fields and cracking phenomena in asphalt materials. Guo et al. [29] applied DIC technology to the low-temperature crack resistance study of fiber–asphalt mixtures and found that basalt fiber had a more significant effect in improving fracture energy and inhibiting crack propagation. Jiang et al. [30] further combined DIC with a modified SCB test to analyze the local deformation response during material healing and fracture. In addition, Gao et al. [31] analyzed the dispersion of basalt fiber in asphalt mixtures and the microscopic mechanism by which it inhibits cracking through microscopic observation, while Stewart et al. [32] showed that DIC can be used to identify the local evolution characteristics during fatigue crack propagation in asphalt materials. More recently, Zielinski et al. [33] combined SCB with DIC and image segmentation techniques to evaluate the fracture properties of asphalt concrete mixes under different aging conditions, and demonstrated that post-peak parameters such as the flexibility index and fracture performance index exhibit the best sensitivity in differentiating mixture performance. These findings indicate that the combination of SCB and DIC technology can simultaneously provide macroscopic fracture parameters and full-field deformation information throughout the loading process, thereby enabling a more systematic understanding of crack development.

Overall, existing research has demonstrated that basalt fiber can enhance the crack resistance of asphalt mixtures. The semi-circular bending test can characterize the macroscopic fracture response, while digital image correlation technology can reveal the local deformation characteristics during crack propagation. However, most current studies focus on optimizing fiber parameters or evaluating performance under single test conditions. There is a significant gap in systematic research on how the coupling effects of gradation structure, asphalt binder and test conditions control the crack propagation behavior and fracture mechanism. Specifically, the maximum nominal particle size alters the skeleton structure, the binder affects energy dissipation and the test conditions change the viscoelastic fracture response of the material. These factors collectively influence basalt fiber–asphalt mixtures, but such studies are scarce. Therefore, this study selected AC-13 and AC-20 gradations and AC-13 asphalt binder. Through SCB tests, DIC technology and crack path analysis, the individual and coupled effects of gradation, binder, loading rate and temperature on the crack resistance and crack propagation behavior of basalt fiber–asphalt mixtures were systematically investigated. The main objective was to quantify the variation patterns of multiple factors on fracture performance, providing experimental references for the application of basalt fiber in asphalt mixtures and related structural design.

## 2. Materials and Methods

### 2.1. Raw Materials

#### 2.1.1. Asphalt Binder

In this study, a modified asphalt with a grade of PG76-22 of styrene–butadiene–styrene (SBS) was used. The penetration, softening point and ductility were tested by the methods specified in JTG-F40: T0604, T0606 and T0605. The penetration was 7.4 mm, the softening point was 81 degrees Celsius and the ductility (at 5 cm per minute and 5 degrees Celsius) was 43 cm. The performance of this asphalt mixture met the requirements of JTG-F40 [34].

#### 2.1.2. Basalt Fiber

**Figure 1:**
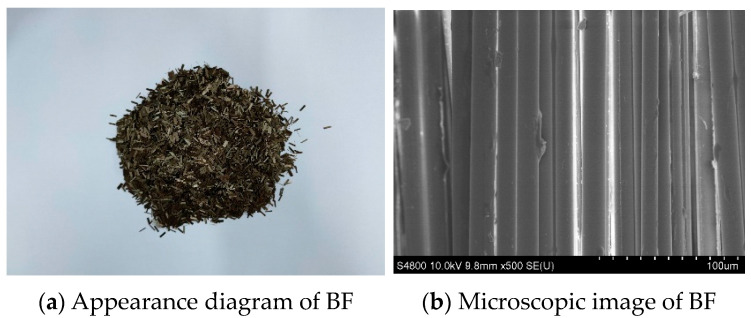
Images of BF.

The length of the base rock fibers used in this study was 6 mm and the diameter was 16 micrometers. The performance of the basalt fibers was tested in accordance with the standard “Fibers for Asphalt Pavement” [35]. The morphology and main physical properties of the basalt fibers are shown in Figure 1 and Table 1, respectively.

#### 2.1.3. Aggregate

In this study, the coarse aggregate particle size was basalt, and the fine aggregate particle size was limestone. The mineral powders were limestone powders. All the aggregates and mineral powders were qualified.

### 2.2. Preparation of Fiber-Reinforced Asphalt Mixture and Mortar

**Figure 2:**
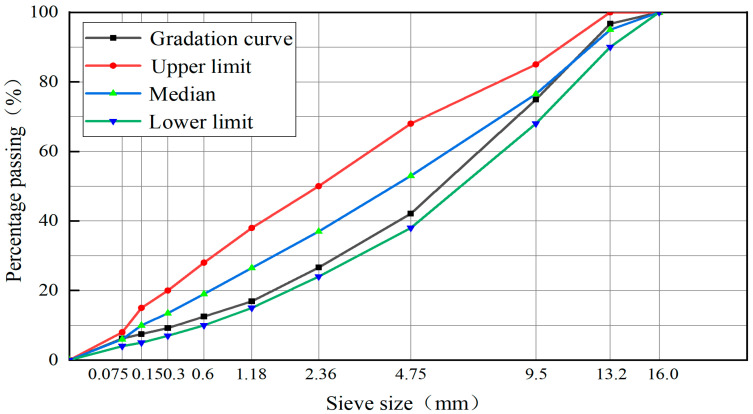
Design gradation curve of AC-13.

**Figure 3:**
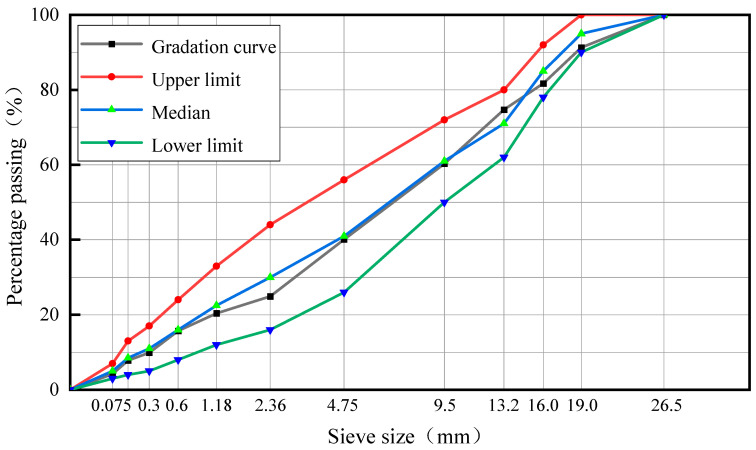
Design gradation curve of AC-20.

Two dense-graded asphalt mixtures, AC-13 and AC-20, were selected in this study, where the numbers denote the nominal maximum aggregate size (NMAS): AC-13 refers to the mixture with an NMAS of 13.2 mm, while AC-20 corresponds to that with an NMAS of 19.0 mm. The mix proportions of both asphalt mixtures were determined via the Marshall approach, and their corresponding aggregate gradation profiles are depicted in Figure 2 and Figure 3. The upper and lower specification limits shown in the figures are based on the JTG F40 standard [34].

During the preparation of the basalt fiber–asphalt mixture, the 6 μm diameter chopped basalt fibers and heated aggregates were first dry-mixed in proportion for 30 s to ensure an even distribution. After the fibers and aggregates were evenly mixed, asphalt was added and mixed for 90 s, followed by the addition of mineral powder for a final mixing.

The target mix proportion design for AC-13 and AC-20 asphalt mixtures was carried out using the Marshall test proportion design method [36]. The test method involved double-sided compaction 75 times, and each group used four test specimens for the test. Thus, the optimal oil-to-stone ratio for the AC-13 gradation was determined to be 5.0% (the percentage of asphalt quality relative to the quality of the aggregates is asphalt), and after adding basalt fiber, the optimal oil-to-stone ratio became 5.2%; for the AC-20 gradation, the optimal oil-to-stone ratio was 4.5%, and after adding basalt fiber, the optimal oil-to-stone ratio became 4.7%.

The gradation of the asphalt mortar is the result obtained by removing the coarse aggregate particle size from the AC-13 aggregate gradation. The design results are shown in Table 2. The asphalt content accounts for 11.77% of the mortar’s mass. Both variants of asphalt mortar samples, measuring 150 mm in diameter, were fabricated via the gyratory compaction technique. The height of the specimens was 150 mm and the void ratio was 1.03% [37].

### 2.3. Test Methods

#### 2.3.1. SCB Test

**Figure 4:**
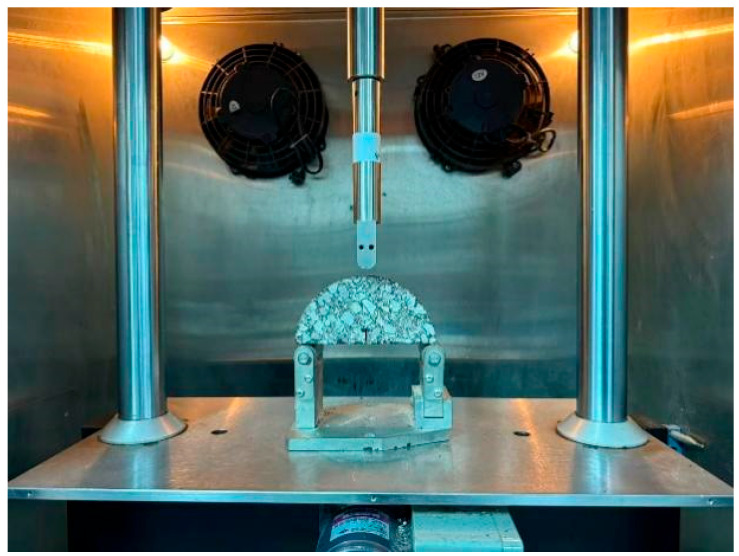
Load diagram of the specimen.

In the test section, parallel samples have all been included. For the Marshall test, each group used four specimens for testing, while in the SCB test, three specimens were used for testing. This makes the test more persuasive. The cylindrical test specimens with a diameter of 150 mm and a height of 250 mm were prepared using a rotary compaction instrument [36]. The SCB test was used to characterize the macroscopic fracture response of the material. Semicircular test pieces, measuring 150 mm in diameter and 50 mm in height, were fabricated by sectioning the initial gyratory-compacted samples. To mimic the behavior of a pre-existing flaw, a 15 mm artificial notch was sawn at the mid-span of the bottom edge. The porosity of the SCB samples of AC-13 and AC-13 BF used in this study was 6.8% and 6.9%, respectively, while the porosity of the SCB samples of AC-20 and AC-20 BF was 6.7% and 6.8%, respectively. At a test temperature of 25 °C, the mixture was maintained for more than 4 h. Using the UTM-25 material testing machine (Controls Group, Liscate, Italy), a vertical loading rate of 50 mm/min was applied to test the crack resistance performance of the asphalt mixture under working conditions with cracks. The testing procedure was executed using a UTM-25 apparatus and the specimen configuration and loading mode are shown in Figure 4. The fracture and flexibility indexes were used to evaluate the SCB-derived fracture parameters of the mixtures, and their calculation formulas are given in Equations (1) and (2). Hassan et al. [38] proposed to use the crack tortuosity coefficient to characterize the crack resistance performance of asphalt mixtures. A larger value indicates better crack resistance of the specimen. Its calculation formula is given in Equation (3).

(1) $$G_{f}=\frac{W_{f}}{\left|m\right|}\times A$$

(2) $$FI=\frac{G_{f}}{\left|m\right|}\times A$$

where $G_{f}$ is the fracture energy, J/m^2^; $W_{f}$ is the work of fracture, J; FI is the flexibility index; $\left|m\right|$ is the absolute value of the post-peak load slope m, kN/mm; and A is the unit conversion and scaling, 0.01.

(3) $$\mathrm{Tortuosity}\mathrm{coefficient}=\frac{L_{m}}{L_{p}}$$

where $L_{m}$ is the length of the crack curve, mm; $L_{p}$ is the projected crack length, mm.

Hofman et al. [39] conducted experiments and numerical simulations to calculate the critical stress intensity factor. When the stress intensity factor at the crack tip reaches the critical value, the crack will undergo instability expansion. This critical value is known as fracture toughness, which is related to the crack size, the geometric characteristics of the component and the load. The critical stress intensity factor K_IC_ can be calculated according to Equations (4)–(6).

(4) $$\sigma_{max}=\frac{4.236F_{max}}{2rt}$$

(5) $$K_{IC}=\sigma_{max}\sqrt{\pi a}f(\frac{a}{r})$$

(6) $$\begin{array}{c} f(\frac{a}{w})=-0.623+29.29(\frac{a}{r})-171.2{\left(\frac{a}{r}\right)}^{2}+457.1{\left(\frac{a}{r}\right)}^{3}\\ -561.2{\left(\frac{a}{r}\right)}^{4}+265.54{\left(\frac{a}{r}\right)}^{5} \end{array}$$

where $\sigma_{max}$ is the maximum stress; $F_{max}$ is the maximum load; and $K_{IC}$ is the critical stress intensity factor.

#### 2.3.2. Digital Image Correlation (DIC)

**Figure 5:**
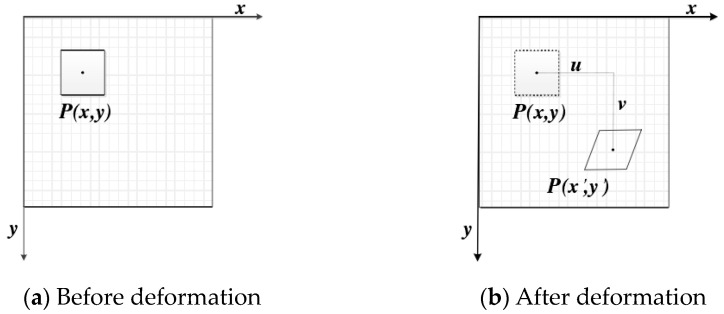
Illustration of the image deformation.

By correlating the gray-level intensities of these regions between the two images obtained from the DIC technology, it is possible to determine the displacement and strain fields of the object, as shown in Figure 5, identifying digital image points P(x, y) and P(x′, y′) before and after the change in the (2M + 1) × (2M + 1) area of the object’s surface. Based on the second-order function [40,41], the relationship can be expressed as Equation (7) [40]. *f_m_* and *g_m_* are the average gray levels of the subregions before and after deformation, which can be calculated by Equation (8) [42].

(7) $$\left\{\begin{array}{l} x^{\prime }=x+u+\frac{\partial u}{\partial x}△x+\frac{\partial u}{\partial y}△y+\frac{\partial^{2}u}{\partial x\partial y}△x△y+\frac{1}{2}\frac{\partial^{2}u}{\partial y^{2}}△y^{2}+\frac{1}{2}\frac{\partial^{2}u}{\partial x^{2}}△x^{2}\\ y^{\prime }=y+v+\frac{\partial v}{\partial x}△x+\frac{\partial v}{\partial y}△y+\frac{\partial^{2}v}{\partial x\partial y}△x△y+\frac{1}{2}\frac{\partial^{2}v}{\partial y^{2}}△y^{2}+\frac{1}{2}\frac{\partial^{2}v}{\partial x^{2}}△x^{2} \end{array}\right.$$

(8) $$\left\{\begin{array}{l} f_{m}=\frac{1}{{\left(2M+1\right)}^{2}}\sum_{x=-M}^{M}\sum_{y=-M}^{M}f(x,y)\\ g_{m}=\frac{1}{{\left(2M+1\right)}^{2}}\sum_{x=-M}^{M}\sum_{y=-M}^{M}g(x^{\prime },y^{\prime }) \end{array}\right.$$

where *u*, *v* are displacements in the *x* and *y* directions; △x, △y is the distance from the point to the center; $\frac{\partial u}{\partial x}$, $\frac{\partial u}{\partial y}$, $\frac{\partial v}{\partial x}$, and $\frac{\partial v}{\partial y}$ are first-order displacement gradients; and $\frac{\partial^{2}u}{\partial x\partial y}$, $\frac{\partial^{2}u}{\partial y^{2}}$, $\frac{\partial^{2}u}{\partial x^{2}}$, $\frac{\partial^{2}v}{\partial x\partial y}$, $\frac{\partial^{2}v}{\partial x^{2}}$, and $\frac{\partial^{2}v}{\partial y^{2}}$ are second-order displacement gradients.

**Figure 6:**
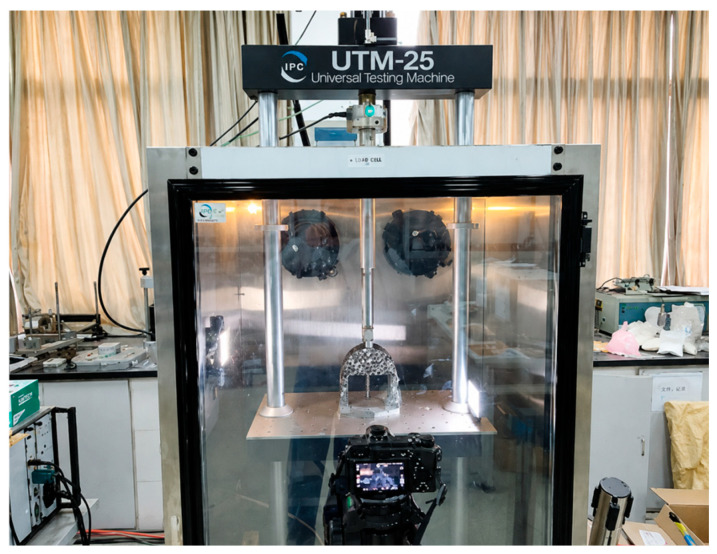
Physical map of the test.

**Figure 7:**
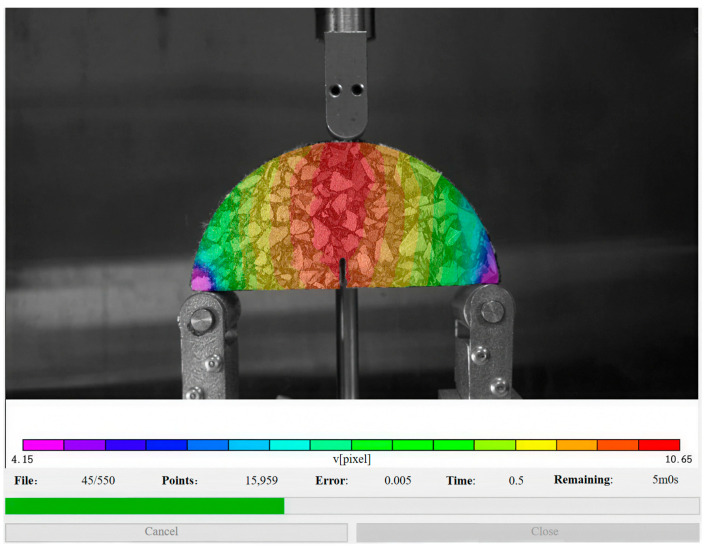
Nephogram of strain.

The specimen surface was polished, wiped clean and air-dried. No paint was needed due to the natural contrast between limestone aggregate and asphalt mortar. A SONY a6400 camera with a polarization filter captured videos at 25 fps with two fill lights, as shown in Figure 6. Frames were extracted using the “ffmpeg” tool within VIC-2D 6.0 software. After selecting the area of interest and scale calibration, displacement and strain data were obtained, as illustrated in Figure 7.

#### 2.3.3. Synchronous Fracture Characterization Method Based on SCB and DIC

**Figure 8:**
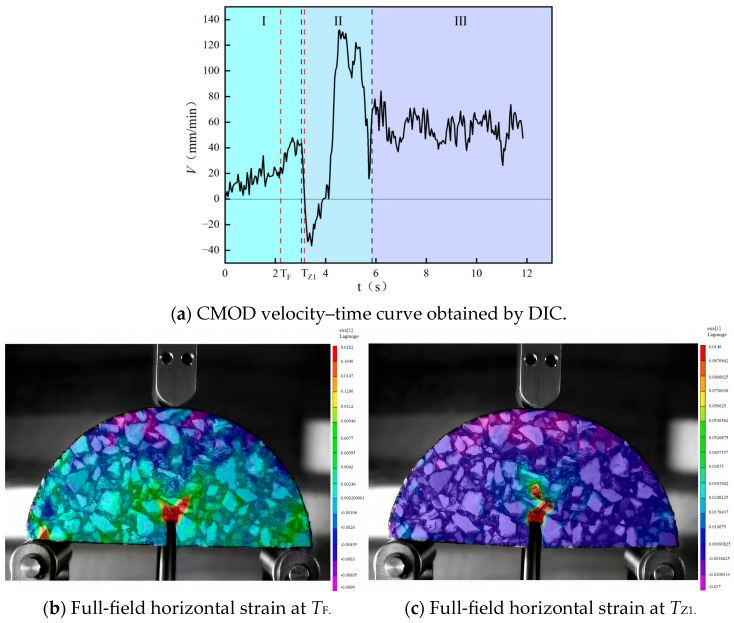
Based on the simultaneous identification of the cracking process of asphalt mixtures by SCB and DIC.

The cracking process was identified by combining SCB with DIC tests, as shown in Figure 8. From the load-displacement graph of the SCB test, the peak load time *T*_F_ can be obtained, as shown in Figure 8a. At the same time, during the same period, the DIC test provided the full-field horizontal strain on the specimen surface, as shown in Figure 8b,c. Combining these three indicators can determine the crack initiation time and the stress–strain development trend.

From Figure 8a,b, it can be seen that when the peak load is reached, the forward horizontal strain is confined to a small area at the top of the pre-cut notch, while the rest of the specimen shows a nearly zero horizontal strain. This indicates that the crack has just formed at the notch top but has not yet extended, corresponding to the smooth ascending segment (Stage I) of the Crack Mouth Opening Displacement (CMOD) speed curve in Figure 8a.

From Figure 8a,c, it can be observed that as loading continues, the CMOD speed steadily increases, marking the transition from Stage I to Stage II in Figure 8a. This transition time is defined as *T*_Z1_. At the same time, the strain shown in Figure 8c indicates that the high-strain region rapidly expands upward along the crack path from the notch top. Therefore, the synchronization of the CMOD speed and strain field indicates that *T*_Z1_ marks the end of microcracks and the beginning of rapid crack propagation. Thus, *T*_Z1_ is used as a quantitative indicator for the crack initiation time in asphalt mixtures.

## 3. Results and Discussion

### 3.1. Effect of Aggregate Gradation on Fracture Performance of Fiber-Reinforced Asphalt Mixtures

#### 3.1.1. Effect of Nominal Maximum Aggregate Size (NMAS)

**Figure 9:**
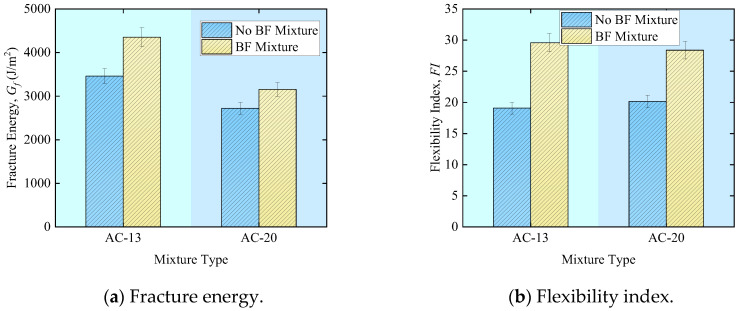
Effect of different nominal maximum aggregate sizes on fracture parameters of asphalt mixtures.

In this section, the legend “No BF Mixture” indicates a mixture without BF, “BF Mixture” represents a mixture with BF and “AC-13 BF” indicates an AC-13 mixture with BF added. Error bars have been added to all the graphs to illustrate the fluctuation deviations of the test data. Figure 9 illustrates the influence of different nominal maximum aggregate sizes on the fracture energy and flexibility index of asphalt mixtures under a loading condition of 25°C and 50 mm/min; these results are the average values obtained from three groups of specimens, with an error of less than 5%. The fracture energy of the AC-13 asphalt mixture is 3459.67 J/m^2^, while that of the AC-20 mixture is only 2721.47 J/m^2^, representing a 27.1% reduction. The explanation could be that the coarse aggregate particle size contains more large-sized aggregates, around which the asphalt mortar film thickness is unevenly distributed, making it easier for stress concentration zones to form at the mortar–aggregate interfaces. However, the observed differences are also associated with the differences in asphalt binder content imposed by the Marshall mix design procedure. AC-13 contains approximately 0.5% higher binder content than AC-20, which likely contributed to a portion of the observed improvements in fracture energy. After incorporating 6 mm basalt fibers, the fracture energy of the AC-13 and AC-20 asphalt mixtures increases to 4352.26 J/m^2^ and 3155.24 J/m^2^, respectively, which is 25.8% and 15.9% higher than that of the control groups. The flexibility index (*FI*) also increased to 29.58 and 28.38. Although the NMAS varies, both the fracture energy and flexibility index of the asphalt mixture and the basalt fiber–asphalt mixture show a decreasing trend, and the reinforcing effect of the fibers on asphalt mixtures with different NMAS differs. The enhancement effect of 6 mm basalt fibers on the fracture energy of AC-13 is better than that on the AC-20. This indicates that the 6 mm crushed fibers are relatively shorter, resulting in a weaker bridging and pulling-out effect on the large particles in AC-20, and thus making it difficult for these fibers to fully exert their reinforcing effect at both ends of the cracks. This finding suggests that a synergistic matching relationship may exist between fiber length and the maximum aggregate size, which is a key consideration in designing high-performance fiber-reinforced mixtures.

**Figure 10:**
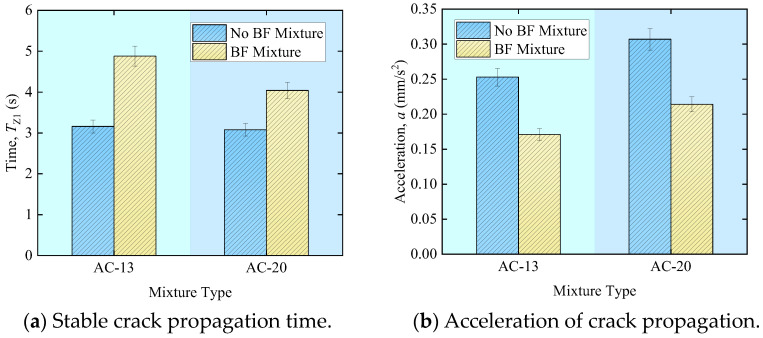
Comparison of micro-dynamic fracture characteristics of asphalt mixtures with different nominal maximum aggregate sizes.

Further observation of the stable crack propagation stage, as listed in Figure 10, shows results that are the average values obtained from three groups of specimens, with an error of less than 5%, which shows that the stable crack propagation time (*T*_Z1_) of the AC-13 asphalt mixture is 3.16 s, while that of AC-20 is 3.08 s, indicating comparable performance. However, after fiber addition, the *T_Z1_* of AC-13 extends to 4.88 s, an increase of 1.72 s, while that of AC-20 only extends to 4.04 s, an increase of 0.96 s. Meanwhile, the crack propagation acceleration shows a similar trend: it decreases from 0.253 mm/s^2^ to 0.171 mm/s^2^ for AC-13 (a 32.4% reduction) and from 0.307 mm/s^2^ to 0.214 mm/s^2^ for AC-20 (a 30.3% reduction). It is worth noting that the initial acceleration of AC-20 is greater, which indicates that once cracking begins, the expansion process will be more intense—this is consistent with the judgment that coarse-grained materials exhibit an uneven stress distribution and local stress concentration.

#### 3.1.2. Asphalt Mortar

**Figure 11:**
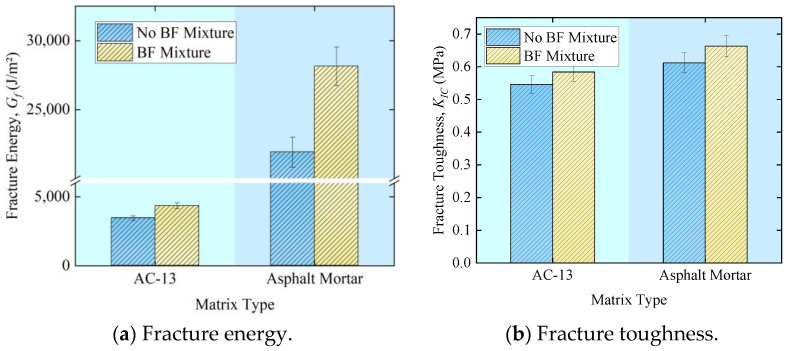
Comparison of fracture parameters between asphalt mixture and asphalt mortar.

To reveal the underlying mechanisms of fiber reinforcement, this study comparatively analyzed the fracture performance of asphalt mixtures and their matrix phase, asphalt mortar, with results shown in Figure 11; these results are the average values obtained from three groups of specimens, with an error of less than 5%. The fracture energy and fracture toughness of asphalt mortar are 21,920.34 J/m^2^ and 0.612 MPa, respectively, which are significantly higher than those of the AC-13 mixture (3459.67 J/m^2^ and 0.546 MPa). This significant difference stems from the fundamental difference in the material structure: eliminating the coarse aggregate particle size will increase the amount of asphalt, thereby causing the disappearance of large particle asphalt adsorption. As a result, it will significantly affect the fracture behavior. It also limits its deformability and introduces a large number of mortar–aggregate interfaces—these are mechanically weak zones that readily become preferential channels for crack initiation and propagation.

After adding BFs, the fracture energy of the asphalt mixture and asphalt mortar increases to 4352.26 J/m^2^ and 28,146.67 J/m^2^, respectively, increasing by 25.8% and 28.4%, respectively. Correspondingly, their fracture toughness increases to 0.584 MPa and 0.663 MPa, increasing by 6.9% and 8.3%. The basalt fibers mainly form a three-dimensional network structure in asphalt mortar, playing the role of supporting stress and delaying the appearance of matrix cracks, thereby significantly improving the crack resistance of asphalt mortar.

### 3.2. Effect of Testing Conditions on Cracking Performance of Fiber-Reinforced Asphalt Mixtures

#### 3.2.1. Loading Rate

**Figure 12:**
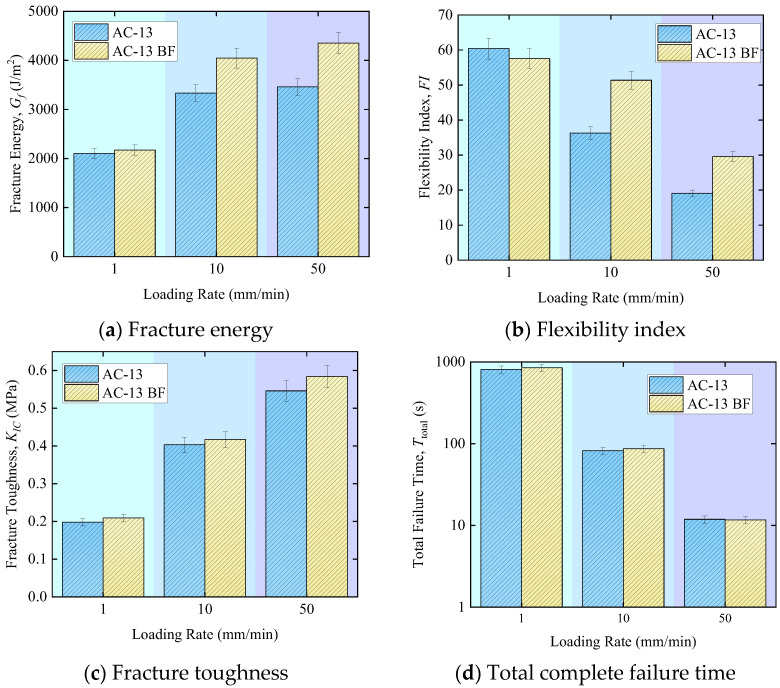
Effect of loading rate on fracture parameters of fiber-reinforced asphalt mixtures.

Figure 12 systematically presents the influence of loading rate on fracture parameters; these results are the average values obtained from three groups of specimens, with an error of less than 5%. As the loading rate decreases from 50 mm/min to 1 mm/min, the fracture energy of the AC-13 asphalt mixture drops sharply from 3459.67 J/m^2^ to 2099.17 J/m^2^, a reduction of 39.3%; meanwhile, the fracture energy of the basalt fiber-reinforced mixture decreases from 4352.26 J/m^2^ to 2172.33 J/m^2^, a reduction of 50.1%. Compared with the fiber-free asphalt mixture, the enhancement amplitude of fracture energy is 25.8%, 21.3% and 3.5%. This means that when the loading rate is extremely low (1 mm/min), the fiber almost loses its reinforcement effect. Regarding *FI*, as the loading rate decreases from 50 to 1 mm/min, the value of the asphalt mixture increases from 19.07 to 60.37, while that of the basalt fiber–asphalt mixture increases from 29.58 to 57.56. On the contrary, the fracture toughness (*K_IC_*) significantly decreases. The asphalt mixture decreases from 0.546 MPa to 0.198 MPa, while the basalt fiber–asphalt mixture decreases from 0.584 MPa to 0.209 MPa. At the same time, the total time for complete failure of the asphalt mixture increases from 11.88 s to 809.61 s, while that of the basalt fiber–asphalt mixture increases from 11.67 s to 847.38 s, showing a significant trend of prolonged failure time. These results indicate that a lower loading rate weakens the crack resistance and fracture toughness of the asphalt mixture, increases its toughness and significantly reduces the crack resistance enhancement effect of basalt fibers; the observed differences are also associated with the differences in asphalt binder content imposed by the Marshall mix design procedure. The loading speed significantly affects the flexibility index (*FI*) of asphalt mixtures. There is a clear inverse relationship between the two. The slower the loading speed, the higher the *FI* value. The fundamental cause of the above phenomenon lies in the viscoelastic nature of asphalt materials. Asphalt is a typical time–temperature-dependent viscoelastic material whose mechanical behavior is strongly governed by the loading rate (or time scale). At low loading rates, the matrix material has sufficient time to undergo molecular chain rearrangement and creep deformation, and the stress at the crack tip is gradually released through viscous flow. Its resistance to cracking is fully exerted, thus resulting in a higher *FI* value. When stress relaxation dominates the material behavior, deformation is primarily controlled by plastic flow, and the degree of stress concentration at the crack tip is significantly reduced. Under this condition, the interfacial bonding and physical anchoring between the fibers and the matrix are also weakened—the fibers have not yet fully developed their bridging effect, while the matrix has already released the stress through plastic deformation; consequently, the fibers can hardly “sense” sufficient stress to activate their reinforcement function. In contrast, at high loading rates, the asphalt matrix exhibits more pronounced elastic behavior, stress relaxation has no time to occur and a high stress concentration rapidly develops at the crack tip, allowing the fibers to be quickly activated at the moment of crack initiation, thereby exerting their bridging and crack-arresting roles.

#### 3.2.2. Testing Temperature

**Figure 13:**
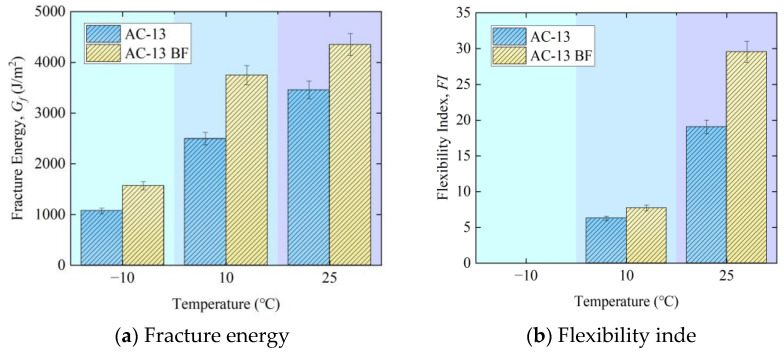
Effect of testing temperature on fracture parameters of fiber-reinforced asphalt mixtures.

Figure 13 presents the systematic evolution of fracture parameters as the temperature decreases from 25°C to 10°C and −10°C; these results are the average values obtained from three groups of specimens, with an error of less than 5%. At −10°C, the asphalt mixture has already cracked, so the FI value cannot be measured. As the temperature drops, the asphalt mixture undergoes a transition from viscoelastic to elastic–brittle behavior. The fracture energy of the asphalt mixture decreases from 3459.67 J/m^2^ at 25°C to 1073.67 J/m^2^ at −10°C, a reduction of as much as 69.0%. Similarly, the fracture energy of the BF-reinforced mixture decreases from 4352.26 J/m^2^ to 1567.33 J/m^2^, a reduction of 64.0%. This indicates that low-temperature environments severely deteriorate the energy dissipation capacity of asphalt mixtures; the observed differences are also associated with the differences in asphalt binder content imposed by the Marshall mix design procedure.

Similar to the trend of fracture energy, the flexibility index decreases significantly with the decrease in temperature: the *FI* of the asphalt mixture decreases from 19.07 at 25°C to 6.29 at 10°C, while the *FI* of the reinforced BF mixture decreases from 29.58 to 7.73; at −10°C, both mixtures exhibit cracking. The synchronous decrease in fracture energy and *FI* reflects the characteristic of low-temperature brittle hardening. As the temperature decreases, the hardness and stiffness of the material increase and the viscoelastic deformation ability is rapidly lost, resulting in a sharp decline in energy absorption capacity, making it more prone to sudden brittle failure.

From the perspective of the fiber-reinforcement effect, the test temperature changed the contribution mechanism of the fibers. In terms of the fracture energy, at 25 °C, 10 °C and −10 °C, the enhancement amplitudes created by basalt fibers are 25.8%, 50.2% and 46.0%, respectively. This indicates that at lower temperatures, the effect of the fibers has been enhanced. On the contrary, the enhancement amplitude of the flexibility index decreased from 55.1% at 25 °C to 22.9% at 10 °C. Both mixtures exhibited brittle fracture at −10 °C. These data reveal an important rule: as the temperature decreases, the extreme hardening of asphalt limits its deformation ability, resulting in a decrease in the *FI* enhancement amplitude. However, the enhancement amplitude of the fracture energy indicates that at low temperatures, the crack resistance protection provided by BF is more significant than in normal temperature environments. In other words, the fibers exhibit their maximum value at the moment when the material is the weakest and most prone to sudden brittle fracture.

### 3.3. Cracking Paths of Fiber-Reinforced Asphalt Mixtures and Mortars

**Figure 14:**
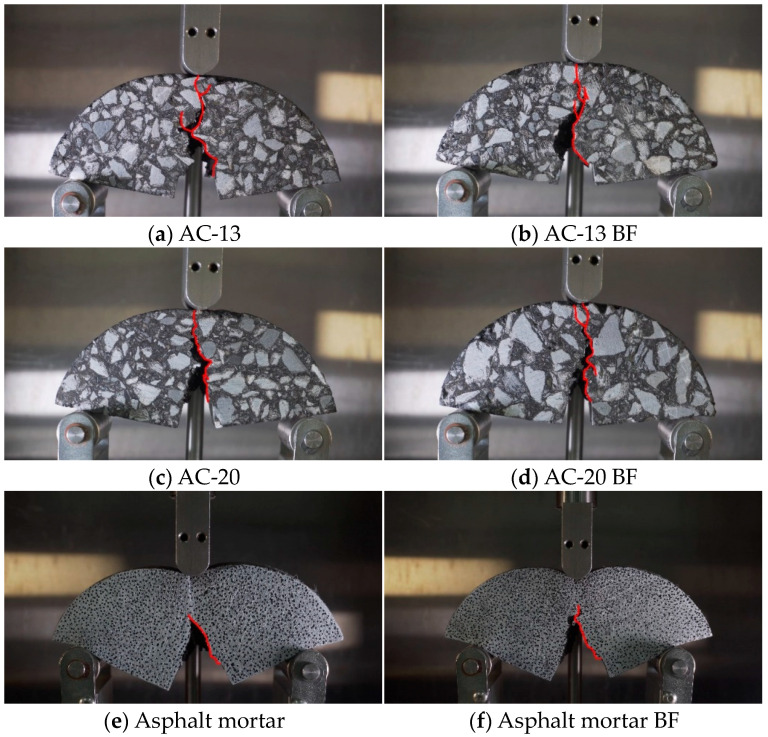
Cracking paths of specimens with different aggregate gradations and matrices under the condition of 25 °C and 50 mm/min.

**Figure 15:**
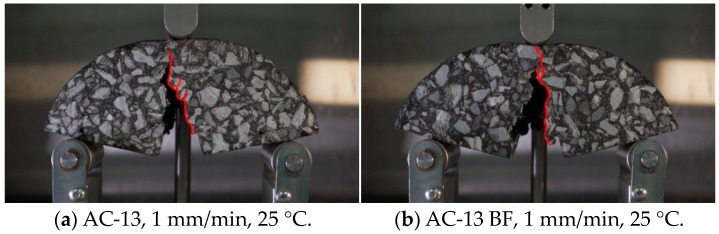

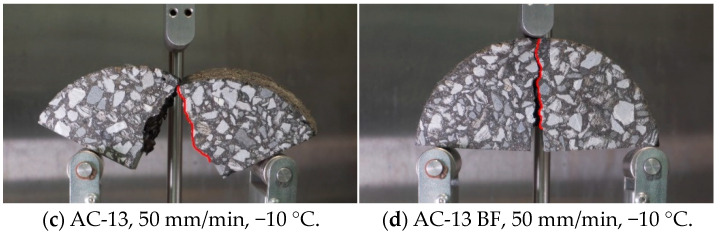
Representative cracking paths of specimens under different loading rates and testing temperatures.

**Figure 16:**
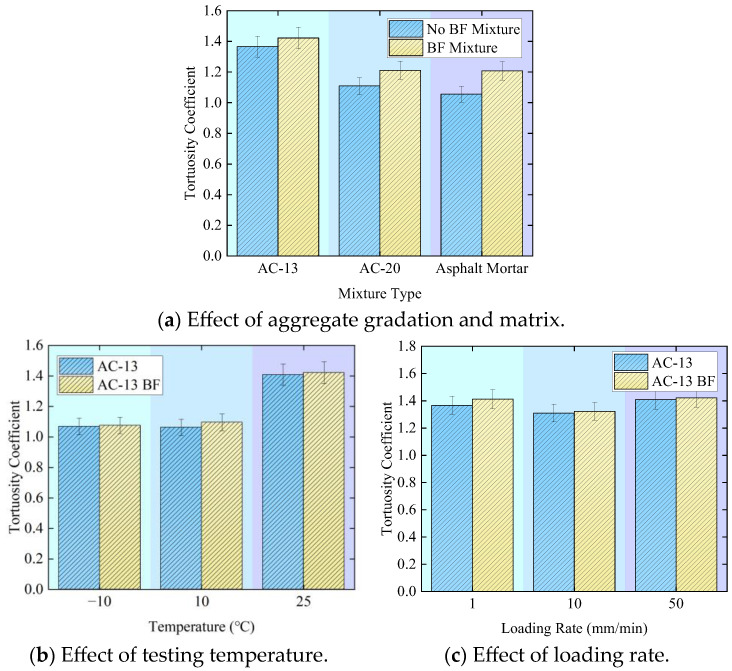
Effect of different testing conditions on the crack tortuosity coefficient of specimens.

Figure 14 and Figure 15 intuitively display the crack propagation paths of specimens under different conditions, while Figure 16 systematically quantifies the crack tortuosity coefficient; these results are the average values obtained from three groups of specimens, with an error of less than 5%.

Regarding path preference, cracks preferentially propagate within the asphalt mortar and along the mortar–aggregate interfaces, rarely penetrating directly through the high-strength limestone aggregates (in Figure 15). This observation is consistent with the fundamental principles of composite fracture mechanics: cracks always follow the path of least resistance to propagation. The fracture toughness of aggregates is much higher than that of asphalt mortar and the interfacial transition zone, so cracks naturally choose to bypass rather than penetrate the aggregates. This phenomenon is particularly pronounced in AC-13, where the fine aggregate particle size features small, densely distributed aggregates, forcing cracks to frequently detour, resulting in a highly tortuous path (tortuosity coefficient of 1.366). In contrast, the AC-20 mixture has larger spacing between coarse aggregates, allowing cracks to propagate in a straighter line through longer mortar zones, with a tortuosity coefficient of only 1.109.

Turning to the effect of fiber incorporation, it significantly influences crack morphology. Under the conditions of 25°C and 50 mm/min, the tortuosity coefficients of AC-13, AC-20 and asphalt mortar increase from 1.366, 1.109 and 1.056 to 1.422, 1.211 and 1.208, representing increases of 4.1%, 9.2% and 14.4%, respectively. This gradient-based enhancement has profound mechanistic implications: fibers can most fully exert their “detour-inducing” effect in a relatively homogeneous medium, where the random distribution of fibers forms a dense mechanical obstacle network, forcing cracks to continuously seek weak zones and frequently deflect. In the mixture, the particle size of the coarse aggregate itself constitutes an inherent obstacle for crack expansion. The addition of fibers provides an additional bridging effect on this basis, making the crack path more tortuous. The combined action of the coarse aggregate skeleton and the fiber-reinforced mortar causes the crack to need to bypass more obstacles during the expansion process, thus consuming more energy.

Temperature exerts a dominant influence on crack morphology (Figure 15 and Figure 16). When the temperature decreases from 25°C to 10°C, the tortuosity coefficient of the fiber-reinforced mixture drops from 1.422 to 1.096. And when it further decreases to −10°C, the coefficient drops to 1.076, stabilizing at an extremely low level at around 1.1, with cracks exhibiting a typical straight-through penetration morphology. This abrupt decrease reflects a fundamental transition in the material’s constitutive behavior: from a viscoelastic detour mode at normal temperatures to a brittle penetration mode at low temperatures. At low temperatures, the matrix loses its deformability, and crack propagation is governed by thermodynamic driving forces rather than by local resistance distribution. Consequently, the “detour-inducing” effect of fibers is completely suppressed. Therefore, in low-temperature environments, one should not rely on the “detour toughening” mechanism of fibers, but rather focus on the “bridging and crack-arresting” function of fibers.

## 4. Conclusions

This study investigated the fracture behavior and crack propagation mechanisms of basalt fiber (BF)-reinforced asphalt mixtures, considering the effects of different gradations, mortar properties and testing conditions. The main findings are summarized as follows:

(1) The reinforcing effect of 6 mm basalt fibers on the fracture energy of asphalt mixtures is influenced by the nominal maximum aggregate size of the asphalt mixture, also associated with the differences in asphalt binder content imposed by the Marshall mix design procedure, showing a greater enhancement in the AC-13 mixture than in the AC-20 mixture.
(2) A consistent enhancement in fracture performance is observed between the asphalt mixture and the asphalt mortar after BF incorporation. Under the same test conditions, the addition of fibers increased the fracture energy by 25.8% for the mixture and by 28.4% for the mortar, while fracture toughness increased by 6.9% and 8.3%, respectively. This suggests that the improvement in the fracture parameters of the mixture is closely related to that of the mortar; the observed differences are also associated with the differences in asphalt binder content imposed by the Marshall mix design procedure.
(3) Testing conditions severely interfere with the efficacy boundaries of fibers; the lower loading rate reduces the reinforcement effect due to viscoelastic stress relaxation. Low temperatures enhance the relative crack resistance efficiency of basalt fibers.
(4) The addition of fibers increases the crack tortuosity coefficient, forcing cracks to change directions and dissipate more energy. However, environmental temperature maintains absolute control over the geometric path.

## Figures and Tables

**Table 1:** Test results of fiber properties.

Item	BF	Test Method
Length/mm	6	Appendix H
Diameter/μm	16	Appendix H
Elongation at break/%	4.83	Appendix S
Tensile strength/MPa	2563	Appendix S
Apparent density/g·cm^−3^	2.53	Appendix J

**Table 2:** Design of asphalt mortar grading.

Aggregate Particle Size/mm	2.36	1.18	0.6	0.3	0.15	0.075
Passing rate/%	100	71.0	49.6	33.8	20.9	10.3

## Data Availability

The original contributions presented in this study are included in the article. Further inquiries can be directed to the corresponding author.
